# The Role of Long Non-coding RNAs in Human Imprinting Disorders: Prospective Therapeutic Targets

**DOI:** 10.3389/fcell.2021.730014

**Published:** 2021-10-25

**Authors:** Tingxuan Wang, Jianjian Li, Liuyi Yang, Manyin Wu, Qing Ma

**Affiliations:** Shenzhen Key Laboratory of Synthetic Genomics, Guangdong Provincial Key Laboratory of Synthetic Genomics, CAS Key Laboratory of Quantitative Engineering Biology, Shenzhen Institute of Synthetic Biology, Shenzhen Institutes of Advanced Technology, Chinese Academy of Sciences, Shenzhen, China

**Keywords:** genomic imprinting, lncRNA, epigenetic regulation, imprinting disorders, UBE3A-ATS, ASO, CRISPR-Cas9

## Abstract

Genomic imprinting is a term used for an intergenerational epigenetic inheritance and involves a subset of genes expressed in a parent-of-origin-dependent way. Imprinted genes are expressed preferentially from either the paternally or maternally inherited allele. Long non-coding RNAs play essential roles in regulating this allele-specific expression. In several well-studied imprinting clusters, long non-coding RNAs have been found to be essential in regulating temporal- and spatial-specific establishment and maintenance of imprinting patterns. Furthermore, recent insights into the epigenetic pathological mechanisms underlying human genomic imprinting disorders suggest that allele-specific expressed imprinted long non-coding RNAs serve as an upstream regulator of the expression of other protein-coding or non-coding imprinted genes in the same cluster. Aberrantly expressed long non-coding RNAs result in bi-allelic expression or silencing of neighboring imprinted genes. Here, we review the emerging roles of long non-coding RNAs in regulating the expression of imprinted genes, especially in human imprinting disorders, and discuss three strategies targeting the central long non-coding RNA *UBE3A-ATS* for the purpose of developing therapies for the imprinting disorders Prader–Willi syndrome and Angelman syndrome. In summary, a better understanding of long non-coding RNA-related mechanisms is key to the development of potential therapeutic targets for human imprinting disorders.

## Introduction

In diploid organisms, most genes are transcribed in an unbiased fashion from both alleles. However, in a small subset of genes, genetically identical alleles can be expressed differentially, a process referred to as ASE. In the mammalian genome, common epigenetic examples of ASE include random X-chromosome inactivation in females (Lee, 2011; Deng et al., 2014), genomic imprinting (Peters, 2014), random MAE (Reinius and Sandberg, 2015), allelic expression of antigen receptor (Bergman and Cedar, 2004; Vettermann and Schlissel, 2010), clustered protocadherin (Chen and Maniatis, 2013), and olfactory receptors (Monahan and Lomvardas, 2015). Imprinted genes are expressed strictly or preferentially from either paternally or maternally inherited alleles (referred to as parent-of-origin) (Barlow and Bartolomei, 2014; Huang et al., 2018; Chen and Zhang, 2020). The ASE of imprinted genes depends on differential epigenetic markings during gametogenesis in germline cells, as opposed to gene sequences. After imprinting patterns become established in mature germlines, genomic imprinting in an individual is maintained until genome-wide erasure of epigenetic modification occurs in gamete precursors.

Genomic imprinting has been described in diverse organisms, including marsupials, flowering plants, and insects (Macdonald, 2012). In the human and mouse genome, genomic imprinting has been extensively observed, indicating the conservation and evolutionary significance of this epigenetic regulatory mechanism. While the expression of 1% of human protein-coding genes is estimated to be regulated via genomic imprinting (Im et al., 2005; Patten et al., 2016; Elbracht et al., 2020), many of these imprinted genes are essential for metabolism, development, and the nervous system (Monk et al., 2019; Tucci et al., 2019). Not surprisingly, dysregulated imprinting is closely associated with a broad spectrum of human developmental defects and genetic disorders, including PWS, AS, BWS, SRS, KOS14, and TS14 (Bian and Sun, 2011; Wan et al., 2017). The association between imprinted genes and the clinical features of these human diseases has also been documented in mouse models through the identification of homologous imprinted gene regions corresponding to the imprinted gene regions implicated in human imprinting disorders (Peters, 2014; Tucci et al., 2019).

Long non-coding RNAs are a subgroup of non-coding RNAs defined as having a length longer than 200 nucleotides, and are extensively expressed among the genome (Derrien et al., 2012; Harrow et al., 2012; Knauss and Sun, 2013). The number of lncRNA genes in the human genome has been estimated at 20,000 to 100,000 (Zhao et al., 2016; Fang et al., 2018; Uszczynska-Ratajczak et al., 2018). This number is greater than the canonical protein-coding genes in the human genome (Southan, 2017). lncRNAs are primarily retained in the nucleus, having short half-lives and a rapid turn-over rate compared to mRNAs (Clark et al., 2012; Derrien et al., 2012; Yoon et al., 2015). lncRNAs can regulate gene expression in at least three ways: at the transcription level by modulating gene transcription and chromatin structure, at the post-transcription level by affecting splicing and stability of RNA, and at the translation level by modulating protein translation (referred to review Statello et al., 2021). In the human and mouse genome, imprinted genes often reside together within clusters (2–20 genes), called imprinted clusters or imprinted domains (Ferguson-Smith, 2011). In mammals, lncRNAs are generally located in imprinted clusters that contribute to the establishment and maintenance of monoallelic expression at a genome-scale and long time-range (Andergassen et al., 2019). Here, we summarize the roles of lncRNAs in the regulation of genomic imprinting using several well-established imprinted clusters as examples. We also discuss how the expression pattern of lncRNAs and their epigenetic regulatory functions are affected in imprinting disorders and some cancers. Three potential strategies have been developed to target the central long non-coding RNA *Ube3a-ATS* for the purpose of therapeutically correcting the PWS/AS locus imprinting disorders. We also discuss the functional mechanisms of imprinted lncRNAs in the regulation of mono-allelic imprinted gene expression and how it could help us understand ASE mechanisms and underlying pathological mechanisms of human imprinting disorders, hopefully inspiring additional efficient therapeutic strategies.

## Genomic Basis of Imprinting

Along with more profound analysis of patient samples and well-established mouse reciprocal crossing models using high-throughput sequencing, the monoallelic expression of imprinted genes has been observed extensively in mice and humans (Tucci et al., 2019). Methylomes and transcriptomes derived from human peripheral blood and various adult tissue samples have been combined to identify imprinted methylation and the distribution of imprinted genes across the genome (Baran et al., 2015; Zink et al., 2018). In order to identify mouse imprinted genes, parents from strains with different genetic backgrounds were crossed to obtain heterozygotic individuals, permitting the discrimination of parent-of-origin-dependent transcriptional effects from sequence-dependent allelic expression (Babak et al., 2008; Wang et al., 2008). Imprinted genes in mice are identified based on SNPs specific to paternal or maternal genetic backgrounds, thus permitting the quantitation and comparison of expression levels from both alleles. To date, around 160 imprinted genes have been identified in the human genome, and 200 in the mouse genome (Tucci et al., 2019; Chen and Zhang, 2020). Sixty three of these imprinted genes are shared, suggesting that mouse models could be helpful for understanding imprinting regulation in humans.

**FIGURE 1:**
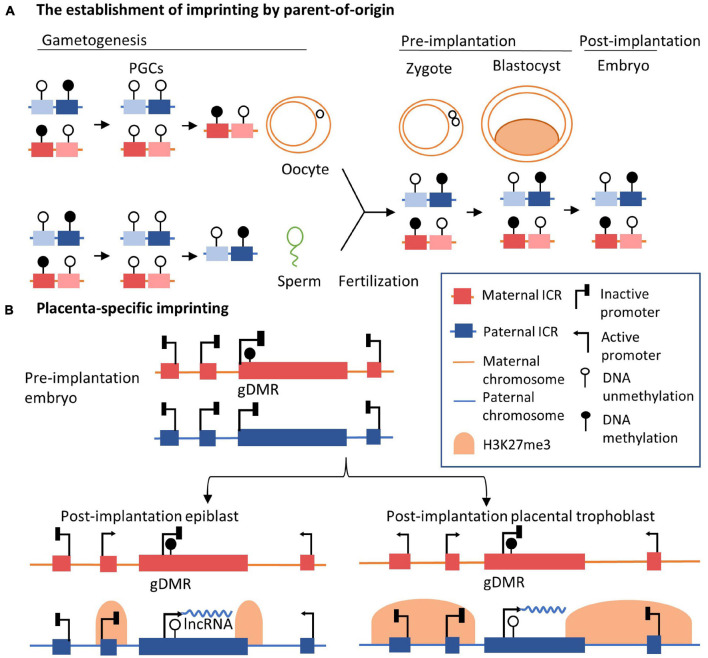
Genomic basis of the regulation of imprinting clusters. **(A)** The inheritance of allele-specific imprinting epigenetic marks across generations. In the early primordial germ cells, epigenetic modifications are erased at a genomic scale before the formation of germline cells. In the germline, parent-of-origin DNA methylation is established, shown as gDMRs. After fertilization and the formation of the zygote, the gDMRs are further maintained. Established imprinting patterns are maintained in blastocyte and somatic cells in adult tissues. **(B)** Imprinting in epiblast and placenta in imprinting loci, such as the *Kcnq1ot1/Kcnq1* or *Airn/Igf2r* loci, is shown. In pre-implantation embryos, DNA methylation is inherited in the gDMR on the maternal allele, such as KvDMR1 of the *Kcnq1ot1/Kcnq1* imprinting cluster. After implantation, the expression of lncRNA on the maternal allele is repressed by DNA methylation in gDMR, allowing the expression of neighbor genes. On the contrary, lncRNA is expressed from the paternal allele, inducing the spreading of H3K27me3 modifications in adjacent regions in the embryonic lineage (epiblast). In extra-embryonic lineage (placental trophoblast), the extended scale of H3K27me3 marks is longer than that seen in embryonic cells. Adjacent genes further away are also silenced on the paternal allele, indicating placenta-specific imprinting, such as *Slc22a18* and *Tssc4* genes in the *Kcnq1ot1/Kcnq1* imprinting cluster. For simplicity, specific gene names are not shown.

In the human and mouse genome, imprinted genes often reside together within imprinted clusters (Ferguson-Smith, 2011). More than 80% of the known imprinted genes in the mouse genome are clustered together in multi-gene ranging in size from less than 100 kb to several megabases (Barlow, 2011). Imprinted lncRNAs located in one imprinted cluster are coordinately controlled by shared regulatory factors, including parent-of-origin-dependent differentially methylated regions (DMRs) and lncRNAs (Peters, 2014). In well-studied imprinted clusters, allele-specific DNA methylation occurs in an independent ICR in the germline, referred to as germline-derived DMRs (gDMRs) or primary DMRs, and persists after fertilization. ICRs in imprinted clusters exhibit parent-of-origin-specific epigenetic modifications, including DNA methylation, governing different expression patterns of parentally inherited alleles (da Rocha and Gendrel, 2019). Around 35 imprinted gDMRs have been identified in the human genome (Monk et al., 2018) and 24 in the mouse genome to date (White et al., 2016). The establishment of gDMRs on paternal or maternal alleles (Figure 1A) is essential for regulating imprinted gene expression in embryonic development (Barlow, 2011; Kelsey and Feil, 2013; Elbracht et al., 2020). In early primordial germ cells, epigenetic marks are extensively erased genome-wide, including DNA methylation and histone modifications. In germline cells, DNA methylation of ICRs is re-established in gametes depending on the parent-of-origin. After fertilization, gDMRs escape secondary global epigenetic reprogramming. DNA methylation information at ICRs of the imprinted regions is retained. In this way, gDMRs of imprinted loci are established robustly during germline development and are resistant to genomic reprogramming after fertilization. Correspondingly, imprinting marks are inherited in a parent-specific manner (Chotalia et al., 2009; Henckel et al., 2012; da Rocha and Gendrel, 2019). gDMRs on the different parent-of-origin alleles are characterized by distinct chromatin configurations, marked with different histone modifications which are corresponding to ‘open chromatin’ and ‘close chromatin’ (Singh et al., 2011; Court et al., 2014; Sanli and Feil, 2015). The allele-specific methylation states of gDMRs are recognized by transcription factors with roles in maintaining parent-of-origin specific expression of the imprinted genes, such as ZFP57 protein (Riso et al., 2016). In total, differential methylation states of gDMRs on parental alleles are essential for the establishment of monoallelic gene expression.

Imprinting control regions govern DNA methylation and chromatin organization in early embryonic and adult lineages, resulting in the persistence of imprinting patterns across generations and their maintenance in adult tissues (Monk et al., 2019). After becoming established at early developmental stages in the germline, gDMRs are maintained in most somatic cells throughout life, resulting in the regulation of allelic expression of imprinted gene clusters. gDMRs also direct the rise of ‘secondary’ DMRs, normally corresponding to repressive chromatin modifications, condensed chromatin structure, and the gene-silencing function of imprinted lncRNAs (Sasaki et al., 1995; Nowak et al., 2011, p. 2; Rao et al., 2014; Tan et al., 2018; Zink et al., 2018). It has been shown that the imprinted expression of some genes is restricted to specific tissues or stages in developmental processes, along with additional allele-specific epigenetic marks further established in somatic cells. The expression patterns of these developmentally expressed imprinted genes are characterized by temporal- and spatial-specific biases (Perez et al., 2015; Andergassen et al., 2017). For example, *UBE3A* and *IGF2* show imprinted expression patterns in specific human brain cell types (Rougeulle et al., 1997; Vu and Hoffman, 1997; Pham et al., 1998; Yamasaki et al., 2003; Li J. et al., 2020). In a study of ASE in diverse tissues from 178 adult post-mortem donors, paternally silenced *IGF2* was reported in the human brain, different from the canonical paternal expression observed in other tissues (Baran et al., 2015). In the mouse E6.5 gastrulating epiblast, it has also been observed that *Igf2r* is expressed from both alleles and further becomes imprinted in the embryonic lineage at the gastrulation stage (Marcho et al., 2015). Besides, the placenta-specific imprinting has been observed, and the underlying mechanism has been well-understood, especially in the potassium voltage-gated channel subfamily Q member 1 (*Kcnq1*)*/Kcnq1* antisense transcript 1 (*Kcnq1ot1*) cluster and the antisense of *Igf2r* non-protein coding RNA (*Airn*)*/Igf2r* cluster (Figure 1B; Sleutels et al., 2002; Andergassen et al., 2019; Hanna, 2020). The establishment of the placenta-specific imprinting initiates by allelic DNA methylation in pre-implantation embryos. In the placenta, the genomic profile of DNA methylation in imprinted DMRs is different, likely the result of an overall different pattern of placenta compared to other tissues (Schroeder et al., 2013). After implantation, the silencing of imprinted genes on the paternal allele in the post-implantation placental trophoblast expands and tends to be larger than the post-implantation epiblast. This expansion of gene silencing is mediated by the spreading of H3K27me3 marks along the paternal chromosome (Calabrese et al., 2015; Andergassen et al., 2017).

## Long Non-Coding RNAs and Their Roles in Regulating the Expression of Imprinted Genes

**FIGURE 2:**
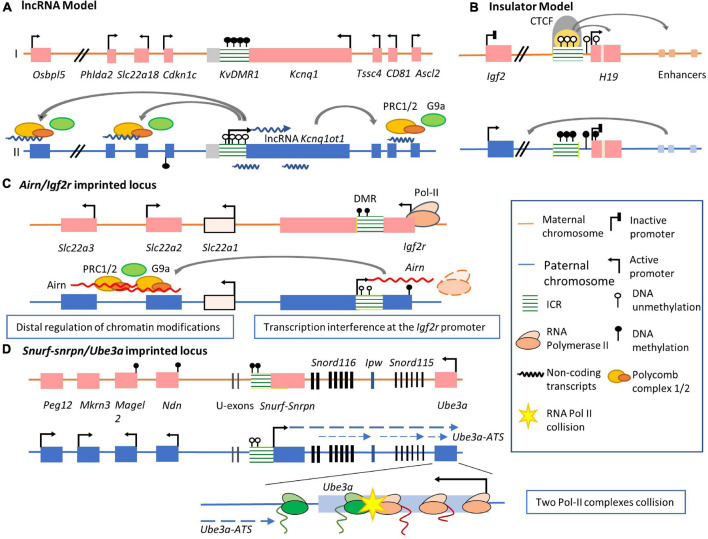
Mechanisms by which imprinted lncRNA regulate allelic expression in imprinted clusters. **(A)** The lncRNA model of imprinted gene expression regulation. In *Kcnq1ot1* imprinted cluster, the ICR is unmethylated on the paternal allele, permitting lncRNA Kcnq1ot1 expression. The expression of this lncRNA recruits the PRC1/2 complex and histone methyltransferase G9a, leading to condensed chromatin and silencing of flanking protein-coding genes. The ICR is methylated on the maternal allele, inhibiting lncRNA expression. The expression of *Kcnq1* and several paternal silenced genes are activated. **(B)** The insulator model of imprinted gene expression regulation. The ICR on the maternal allele is unmethylated. CTCF binds to the maternal ICR and functions as an insulator to block *Igf2* access to its distal enhancers. In contrast, the ICR on the paternal allele is methylated, preventing binding of CTCF. The expression of *Igf2* is activated via enhancer regulation. **(C)** lncRNA *Airn* in *Airn/Igf2r* locus function in two distinctive mechanisms. On the one hand, methylated DMR on the maternal allele inhibits *Airn* expression, allowing access of transcription factors to the *Igf2r* promoter. The paternal DMR is unmethylated, permitting *Airn* transcription. *Airn* overlaps with the promoter of *Igf2r* and inhibits *Igf2r* expression. On the other hand, *Airn* transcripts recruit PRC2 complex to distal genes, such as *Slc22a3* and *Slc22a2*, where they silence expression. *Slc22a1* is a biallelic expressed protein-coding gene between distal regulated imprinted genes and Igf2r gene loci. **(D)** In Snurf-*Snrpn/Ube3a* imprinted cluster, the transcription of *Ube3a-ATS* starts from the exon upstream of the *Snurf-Snrpn* gene on the paternal allele. A group of non-coding RNAs are expressed, including *Snord116* and *Snord115* sno-lncRNAs and SnoRNAs. The elongation of this lncRNA overlaps with the *Ube3a* protein-coding region. A collision occurs between the converging elongation complexes of *Ube3a-ATS* and *Ube3a* resulting in the failure of *Ube3a* transcription elongation. By contrast, on the maternal allele, the ICR of *Snurf-Snrpn*/*Ube3a* cluster is methylated in the brain. G9a is recruited to the methylated DMR. This G9a accumulation leads to condensed chromatin and the silencing of flanking imprinted genes near the *Snurf/Snrpn* gene region. Consequently, the maternal *Ube3a* allele is expressed.

Two major mechanisms have been described to explain the regulation of the gene expression within an imprinted cluster (Lee and Bartolomei, 2013; Barlow and Bartolomei, 2014; Chen and Zhang, 2020). The first model is the lncRNA model, which may be more common. In this model, imprinted lncRNAs regulate imprinted gene expression. In the lncRNA model, imprinted lncRNAs intimately associate with ICRs. Imprinted lncRNAs are characterized by their capacity to silence imprinted genes in the same cluster (Rao et al., 2014; Kanduri, 2016; Tan et al., 2018; Zink et al., 2018; Tucci et al., 2019). As illustrated by the *Kcnq1/Kcnq1ot1* imprinted cluster (Figure 2A), actively expressed imprinted lncRNA *Kcnq1ot1* on the paternal allele can silence multiple imprinted genes bidirectionally along their located gene region (Pauler et al., 2012). In contrast, a maternally methylated ICR on the paternal directly inhibits *Kcnq1ot1* and its silencing effects, leading to the released expression of imprinted genes from the silencing by *Kcnq1ot1*. Another model, the insulator model is identified in other imprinted regions, in which parental allele-specific epigenetic differences at ICRs contribute to topological alternations of imprinted gene regions, inducing gene silencing or activation of specific alleles. This model is mainly applied to explain how imprinted genes in the insulin-like growth factor 2 (*Igf2*)*/H19* locus are mechanistically regulated (Figure 2B; Kaffer et al., 2000). *H19* is a maternally expressed lncRNA (Bartolomei et al., 1991; DeChiara et al., 1991; Ferguson-Smith et al., 1991). The zinc-finger protein CTCF binds to the unmethylated maternal ICR and creates topologically associating domain boundaries, blocking *Igf2* access to the enhancer like an ‘insulator’ (Schoenherr et al., 2003; Gómez-Marín et al., 2015). On the paternal allele, methylated ICR prevents CTCF binding and leads to secondary methylation of the *H19* promoter and therefore silencing of lncRNA expression. The enhancers are then accessible to *Igf2*, permitting paternal-allele expression of *Igf2* (Thorvaldsen et al., 1998; Chen and Zhang, 2020). Different from the lncRNA model, imprinted lncRNAs *H19* in the insulator model are not the key regulation elements or whether imprinted lncRNAs affect other genes are not clear.

Here, we discuss on the role of imprinted lncRNAs in epigenetic regulation in the more common model, lncRNA model (Kopp and Mendell, 2018). lncRNA functions can be characterized based on their specific subcellular locations and interactions with DNA, RNA, and proteins, regulation of chromatin structure, expression of nearby and distal genes, RNA post-transcription modification, or mRNA translation (St Laurent et al., 2015; Kopp and Mendell, 2018; Statello et al., 2021). Imprinted lncRNAs range from 1.9 to 1,000 kb in length (Guenzl and Barlow, 2012) and regulate the expression of adjacent imprinted genes *in cis* through interacting with promoters and transcription factor binding sites, modifying chromatin status, or affecting higher-order structures (Barlow, 2011). Two major functional mechanisms of imprinted lncRNAs in the regulation of imprinted gene expression are hypothesized: interacting with promoters or enhancers of nearby target genes to affect transcription initiation, or overlapping imprinted gene regions, covering the gene body, and regulating the chromatin state of adjacent gene regions. We will also discuss the mechanisms underlying the regulation of imprinted gene expression by imprinted lncRNAs using well-characterized imprinted clusters as examples.

### Transcriptional Interference

#### Inhibition of Transcriptional Initiation

Transcription of imprinted lncRNAs often overlaps with the promoters or enhancers of imprinted genes and influences their transcription (Lee and Bartolomei, 2013). These imprinted lncRNA transcripts often interfere with the transcription machinery of nearby imprinted genes, influencing the recruitment of transcription factors at their promoters (Latos et al., 2012). Based on an analysis of lncRNA and DNA binding in imprinting clusters from multiple mammalian species, it was suggested that the binding of lncRNAs to promoters of imprinted genes may be common (Liu et al., 2017). The *Airn/Igf2r* imprinted cluster in the mouse genome is a well-studied example (Figure 2C; Latos et al., 2012). On the paternal allele, the transcription profile of *Airn* initiates from its promoter embedded within the ICR in a direction antisense to the transcription of the *Igf2r* gene (Latos and Barlow, 2009). It was noted that intragenic truncations of the endogenous lncRNA *Airn* in embryonic stem (ES) cells that do not include the overlapping region are unable to silence the *Igf2r* paternal allele, thus demonstrating that inhibition of RNA polymerase II recruitment to *Igf2r* promoter region does not depend on the overlap between *Airn* transcription and the promoter (Sleutels et al., 2002; Latos et al., 2012; Santoro and Pauler, 2013). Furthermore, during ES cells differentiation, *Airn* expression was also necessary and sufficient to silence *Igf2r* (Santoro et al., 2013). The overlapping regions between *Airn* transcription and *Igf2r* promoter and its gene body instead of *Airn* lncRNA products themselves lead to silencing of *Igf2r* expression.

#### The Disturbance of the Transcriptional Elongation

Another mechanism involves a collision between the converging elongation complexes of imprinted lncRNA and imprinted genes, leading to transcription stalling, premature termination, and subsequent degradation of the imprinted gene transcript (Hao et al., 2017). An example is the *UBE3A/UBE3A-ATS* imprinted domain on human chromosome 15q11-13, in which imprinted genes, including *MAGEL2, NDN, SNRPN, SNORD115*, and *SNORD116*, are silenced on the maternal allele (Horsthemke and Wagstaff, 2008). In contrast, *UBE3A*, which encodes an E3 ubiquitin ligase, is expressed from the maternal allele, especially in neurons in the brain. The homologous imprinted locus in mice has also been identified and studied, locating at a syntenic loci chromosome 7qC (Yang et al., 1998; Figure 2D). In this imprinted cluster, the ICR embedded within the *Snurf-Snrpn* gene is unmethylated on the paternal allele. In mouse neurons, *Ube3a-ATS* lncRNA is expressed specifically from its promoter embedded in the unmethylated ICR (Yin et al., 2012; Meng et al., 2013). Notably, the *Ube3a* promoter region is not methylated differently like *Ube3a-ATS*. This, combined with the observation that *Ube3a-ATS* transcription initiates from an exon region upstream of the *Snurf-Snrpn* gene and elongates approximately 1,000 kb as far as the intronic region of *Ube3a* between exons 4 and 5 (Landers et al., 2004; Lewis et al., 2019), it was hypothesized that the two opposing polymerases of *Ube3a* and *Ube3a-ATS* collide (Figure 2D). This transcriptional collision may lead to premature termination of *Ube3a* transcription inside its exon region on the paternal chromosome. In neurons from the monoallelic genetically engineered mouse model with the transcription of paternal *Ube3a-ATS* allele being terminated, *Ube3a* allele expression was activated on the paternal allele (Meng et al., 2013), resulting in increased expression comparable to maternal *Ube3a* (Meng et al., 2012). In cultured AS mouse neurons with biallelic silenced *Ube3a* expression, Antisense oligonucleotides (ASOs) targeting *Ube3a-ATS* rescued the expression of *Ube3a* efficiently (Meng et al., 2015). Consistently, in human induced pluripotent stem cells (iPSC)-derived neuron cells with biallelic silenced *UBE3A* expression, ASOs targeting *UBE3A-ATS* lncRNA transcripts lead to transcriptional termination by displacement of RNA Polymerase II, releasing the transcription of *UBE3A* (Germain et al., 2021). Recently, in human iPSCs, both sufficient expression of *UBE3A-ATS* lncRNA and two newly identified boundary elements were located inside the *IPW* gene and the *PWAR1* gene (Martins-Taylor et al., 2014; Hsiao et al., 2019). These two genes are located between *SNORD115* and *SNORD116.* In human iPSCs with the boundary elements deleted using gene editing technology, the expression of *UBE3A* was not silenced by up-regulated *UBE3A-ATS* expression (Hsiao et al., 2019). Mapping RNAPII density showed that reduced active RNAPII across the 3′ half of *UBE3A* corresponding to silenced UBE3A. These results together further support the hypothesized collision between *UBE3A-ATS* and *UBE3A* transcription complexes, leading to premature termination of the latter. In summary, the overlap between *Airn* and *Igf2r* promoter region disrupts the initiation of *Igf2r* transcription, while *Ube3a-ATS* silences the expression of *Ube3a* by disturbing its transcriptional elongation.

### Chromatin Modification

Another lncRNA-related imprinting mechanism involves coating the bidirectionally flanking chromosomal region and recruiting repressive chromatin modification factors (Lee and Bartolomei, 2013; Sanli and Feil, 2015; Statello et al., 2021). The interactions between lncRNAs and these chromatin factors facilitate transcriptional silencing of target genes. The repressive chromatin-modification factors methylate DNA and produce histone modifications resulting in condensed chromatin structure and repressed gene expression. Among well-known repressive chromatin-modification factors, PRCs bind and spread across targeted chromatin facilitated by lncRNAs (Kotzin et al., 2016; Marín-Béjar et al., 2017). lncRNAs, genome structures, and CpG islands are essential factors in recruiting these PRCs, which have the capacity to catalyze lysine 119-mono-ubiquitinated histone H2A (H2AK119ub1) and H3K27me3 to repress gene expression through chromatin compaction and antagonization of transcriptional activators (Schwartz and Pirrotta, 2013; Simon and Kingston, 2013; Calabrese et al., 2015; Pintacuda et al., 2017; Colognori et al., 2019; Schertzer et al., 2019; Gil and Ulitsky, 2020; MacDonald and Mann, 2020). In genomic imprinting, some imprinted lncRNAs can bidirectionally direct repression of flanking neighbor imprinted gene region, such as *KCNQ1OT1* lncRNA. Some lncRNAs can target distal gene regions in the same imprinted clusters they locate, such as *Airn*.

#### Locally Recruiting Condensed Chromatin Structure to Neighbor Gene Region

The *Kcnq1/Kcnq1ot1* ICR, also known as KvDMR1 (*KvLQT1* differentially methylated region 1), with the embedded lncRNA *Kcnq1ot1* promoter, is unmethylated on the paternal allele (Figure 2A; Lee et al., 1999, p. 1; Smilinich et al., 1999; Beatty et al., 2006; Ager et al., 2008). lncRNA *Kcnq1ot1* transcripts from the promoter region recruit several epigenetic factors such as the Polycomb group proteins RING1B (Polycomb Repressive Complex 1, PRC1), EZH2 (PRC2), and histone methyltransferase euchromatic histone lysine *N*-methyltransferase-2 (EHMT2 or G9a) to neighboring gene regions, forming repressive histone modifications such as H3K27me3 and H3K9me2 (Figure 2A II; Umlauf et al., 2004; Pandey et al., 2008). The chromatin state around the flanking regions of this lncRNA becomes condensed and results in silencing of flanking multi-protein coding genes such as *Cdkn1c*, *Slc22a18*, and *Tssc4*. On the maternal allele, DNA methylation of KvDMR1 silences the activation of the *Kcnq1ot1* promoter and represses the transcription, releasing the transcription of neighboring genes.

#### Recruiting Chromatin Modification Factors to Distal Imprinted Genes

A typical example of imprinted lncRNA regulating distal imprinted genes through epigenetic silencing is *Airn* and recruitment of PRCs in the placenta (Figure 2C; Latos et al., 2012; Lee and Bartolomei, 2013). As mentioned before, the transcription of *Airn* represses the expression of flanking imprinted gene *Igf2r* by transcriptional interference of the overlapping *Igf2r* promoter without repressive chromatin modification involved. In contrast, distal imprinted genes, such as *Slc22a2* (about 100 kb to *Airn* locus) and *Slc22a3* (about 300 kb to *Airn* locus), are also silenced by *Airn* in the extra-embryonic lineage, where *Airn* mediates the recruitment of PRC1 and PRC2 to distal targets on the paternal alleles (Terranova et al., 2008; Zhao et al., 2010; Schertzer et al., 2019). Recently, Airn was found to silence *Slc22a3* in mouse trophoblast stem cells (Andergassen et al., 2019). Allele-specific chromosome conformation capture studies have suggested that *Airn* transcription throughout the enhancer of *Slc22a3* may silence *Slc22a3* expression by disrupting its promoter-enhancer interactions. However, with monoallelic deletion of the entire *Airn* gene, no essential enhancers for the distal silenced genes were found in the *Airn* gene region. Nonetheless, it has also been shown that *Airn* lncRNA is enriched on the *Slc22a3* promoter together with an H3K9 dimethylase, G9a (Nagano et al., 2008). These results illustrate that *Airn* may target the promoters of distal imprinted genes by recruiting PRCs and G9a. The enrichment of these histone modification factors may lead to condensed chromatin in distal imprinted regions and silence imprinted genes.

## The Role of Imprinted Long Non-Coding RNAs in Human Imprinting Disorders and Cancer

Long non-coding RNAs play essential roles in many biological processes and are related to various human diseases. Altered expression of imprinted loci has been linked to various neurodevelopmental disorders and cancers (Schaller et al., 2010; Huang et al., 2011; Riordan et al., 2013; Peters, 2014). Since imprinted regions are inherited in a parent-of-origin way, defects in one allele may be sufficient to lead to imprinting disorders (Kopp and Mendell, 2018). More specifically, silencing of parentally expressed imprinted genes can lead to the ultimate loss of its expression. Under abnormal conditions, DNA methylation status, allelic expression, and the biological functions of imprinted lncRNAs may be affected. These alterations may relate to human imprinting disorder-related disease phenotypes (Lee and Bartolomei, 2013). Here, we examine several well-studied imprinting disorders and emphasize the roles of imprinted lncRNAs in pathophysiological processes of imprinting-related diseases and cancers.

### Common Molecular Mechanisms of Imprinting Disorders

**FIGURE 3:**
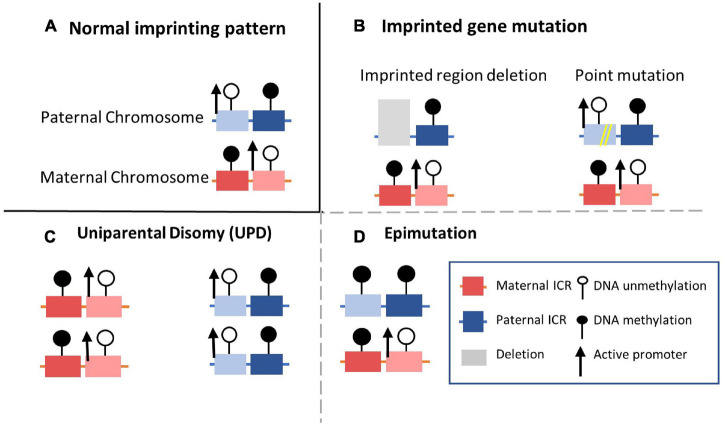
Four common molecular mechanisms of imprinting disorders. **(A)** The normal state of the established DMR methylation pattern on the maternal and paternal alleles. **(B)** Imprinting disorder can be caused by copy number variations with imprinted cluster located such as duplication or deletion. Point mutations (single nucleotide polymorphisms) occurring in imprinted genes could also influence normal functions. **(C)** Both alleles are inherited from same parent-of-origin. In the case shown here, for example, maternally inherited alleles are duplicated without paternal allele participation. **(D)** Epimutations of DNA modification condition can disturb normal imprinting pattern without alterations in DNA sequences of the imprinted region. For example, DNA methylation of the imprinted gene on paternal allele are hypermethylated and silenced on both alleles.

Appropriate expression patterns of imprinted genes are important to growth and development. Correspondingly, imprinting disorder-related human diseases can be caused by genetic or epigenetic abnormalities on paternally or maternally inherited alleles (Lee and Bartolomei, 2013). Several common molecular mechanisms behind imprinting disorders have been defined, including molecular changes or genetic abnormalities, UPD, and epigenetic alterations (Figures 3A–D; Soellner et al., 2017; Carli et al., 2020). Firstly, genetic alterations, including SNPs and copy number variants on one imprinting allele, can affect imprinting (Figure 3B). Another mechanism is UPD, in which the inheritance of two copies of chromosomes or chromosomal regions are both from either the paternal or maternal allele, resulting in synchronous expression or silencing (Figure 3C; Robinson, 2000). Different from genetic alterations, epigenetic changes known as epimutations in DNA or histone modification without obvious genetic mutations have also been documented in imprinting disorders (Figure 3D; Horsthemke, 2010). Hypermethylation at imprinted DMRs can silence the active allele of the original monoallelic expressed imprinted genes. In contrast, hypomethylation can result in overexpression of the original silenced allele. Epimutations can arise randomly or be driven by their environment during the inheritance of germline epigenetic imprinting marks. DNA methylation in DMRs can thus be abnormally inherited in the absence of genetic sequence alterations (Robertson, 2005). Moreover, as with molecular or genetic alterations, epimutations can be permanently maintained in somatic tissues for life and cause developmental phenotypes (Ioannides et al., 2014; Gillessen-Kaesbach et al., 2018; Monk et al., 2019). Besides the imprinted disorder caused by variations in a single imprinted gene, imprinting disorders with epigenetic alterations at loci across the genome have also been observed in many imprinting diseases, referred to as MLID (Horsthemke, 2010; Fontana et al., 2018). Instead of changes at specific genetic loci, MLID may be caused by a globally disturbed imprinting inheritance process across the genome. However, since current research is mostly limited to a subset of imprinted genes and the mosaic character of MILD (Azzi et al., 2014; Eggermann et al., 2021), the role of MLID in imprinting disorders is still poorly understood.

### Congenital Imprinting Disorders and Related Imprinted Long Non-coding RNAs

Molecular disturbances, like loss or gain of methylation at ICRs, and subsequent loss or gain of imprinted gene expression have been described in various congenital human disorders. The frequencies of different molecular abnormalities vary among imprinting disorder-related diseases (Eggermann et al., 2015). More details about typical clinical syndromes and the pathological mechanisms are summarized in Table 1. However, details underlying imprinting disorder mechanisms and how they might impact adult neurobiology and developmental processes remain to be clarified. Fortunately, multiple mouse models of human imprinting disorders have been generated based on genomic conservation in most imprinted clusters. Strong correlates have been shown between these two genomes in imprinting loci, imprinting disorder phenotypes, and underlying molecular mechanisms (Perez et al., 2016). Spatial- and temporal-specific expression of allele-specific genes have been observed in several imprinting clusters in humans and mice. Here, imprinting disorders in three well-studied imprinting clusters are introduced as examples to demonstrate the roles of imprinted lncRNAs in imprinting-related congenital human disorders.

**TABLE 1:** Human imprinting disorder-related diseases.

**Genomic location**	**Imprinted cluster/lncRNA**	**Imprinting disorder diseases**	**Clinical syndromes**	**Molecular mechanisms**	**Prevalence in population**	**References**
Chromosome 15q11-13	*SNUPF-SNRPN/UBE3A* (imprinted lncRNA: *UBE3A-ATS*)	Prader–Willi syndrome (PWS) (OMIM #176270)	Obesity, reduced muscle tone, diminished swallowing and suckling, infantile hypotonia and hypogonadism, intellectual disability	Deletion the imprinted loci on the paternal allele (70–75%); Maternal UPD of chromosome 15 (20–25%); Epimutations of the DNA methylation at ICR 2%); Small deletions within the ICR (<0.5%)	1/25.000–1/10.000	Buiting et al. (1995), Buiting (2010), Fontana et al. (2017), Elbracht et al. (2020)
		Angelman syndrome (AS) (OMIM #105830)	Developmental delay, intellectual disability, absence of speech, microcephaly, seizures, specific excitable demeanor	Deletion of 15q.11–13 region on the maternal chromosome (70–75%); Point mutation in *UBE3A* gene (10%); Paternal UPD (3–7%); *SNURF* ICR loss of methylation (2–3%)	1/20.000–1/12.000	Buiting (2010), Eggermann et al. (2015), Elbracht et al. (2020)
Chromosome 11p-15.5	*H19/IGF2*; *KCNQ1OT1* (Imprinted lncRNA: *H19*) *H19/IGF2*	Beckwith–Wiedeman syndrome (BWS) (OMIM #130650)	Neonatal macrosomia, postnatal overgrowth, placental mesenchymal dysplasia, Tendency to embryonal tumors, cancer predisposition	Paternal UPD of chromosome 11p15.5 (20% to 25%); *KCNQ1OT1*-ICR loss of methylation (50%); H19/IGF2-ICR gain of methylation (5%); *CDKN1C* point mutations (5%); Cluster copy number variation (2–4%)	1/15.000	Eggermann et al. (2016), Mussa et al. (2016), Õunap (2016), Kalish et al. (2017)
		Silver–Russel syndrome (SRS) (OMIM #180860)	Severe intrauterine growth restriction (IUGR), postnatal growth failure with no catch-up, body hemihypoplasia, relative macrocephaly with triangular face, fifth finger clinodactyly and characteristic triangular face, lower birth weight	Loss of methylation at ICR on the paternal allele (40–60%); Maternal UPD of chromosome 7 (5–10%)	1/100.000–1/75.000	Cytrynbaum et al. (2016), Eggermann et al. (2016), Õunap (2016), Wakeling et al. (2017)
Chromo-some 14q32.2	*MEG3/DLK1* (Imprinted lncRNA: *MEG3*)	Kagami–Ogata syndrome (KOS14) (OMIM #608149)	Polyhydramnios, placentomegaly, poor sucking and hypoventilation in the neonatal period, abdominal wall defects, a distinctive facial appearance, small bell-shaped thorax, coat-hanger ribs	Paternal UPD (65%); Microdeletion affecting the maternal 14q32.2 imprinted region (20%); Hypermethylation of the ICR (15%)	<1 in 1,000,000	Beygo et al. (2015), Kagami et al. (2015), Ogata and Kagami (2016), Prasasya et al. (2020)
		Temple syndrome (TS14) (OMIM #616222)	IUGR, PNGR (postnatal growth restriction), hypotonia and motor delay, feeding difficulties in infancy, truncal obesity, scoliosis, precocious puberty, small feet and hands	*MEG/DLK1* ICR loss of methylation (61%); Maternal UPD (29%); Deletion in imprinted region (10%)	<1 in 1,000,000	Ioannides et al. (2014), Gillessen-Kaesbach et al. (2018), Prasasya et al. (2020)

#### *UBE3A-ATS* in Prader–Willi Syndrome and Angelman Syndrome

**FIGURE 4:**
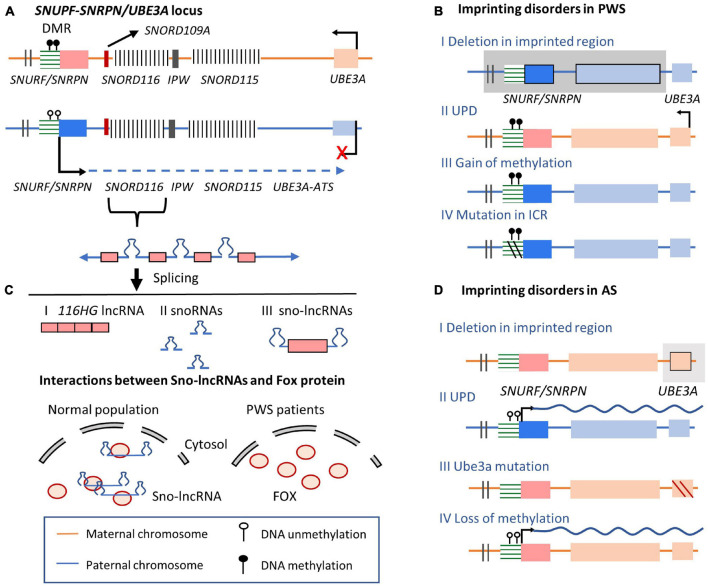
*SNUPF-SNRPN/UBE3A* imprinted cluster on human chromosome 15 and related imprinting disorders. **(A)** Allelic expression pattern in *SNUPF-SNRPN/UBE3A* locus. On the maternal allele, the methylation of ICR silences the expression of *UBE3A-ATS*, permitting *UBE3A* expression. On the paternal allele, *UBE3A-ATS* is expressed from the *SNURF* gene region, overlapping the exon region of *UBE3A* gene. On the paternal allele, lncRNA host transcript are processed to give rise to snoRNAs (*SNORD115* and *SNORD116*), lncRNAs (*116HG*, *115HG*, and *UBE3A-ATS*). Three different spliced non-coding transcripts are produced from *SNORD116* gene locus, including *116HG* lncRNA, snoRNAs, and sno-lncRNAs. *SNORD116* sno-lncRNAs with snoRNAs on two ends are produced after splicing. **(B)** Imprinting disorders occur in PWS. PWS-related molecular alterations in *UBE3A* imprinted gene cluster. Line I indicates deletion in the imprinted region; Line II shows double maternal alleles are inherited, losing the paternal copy; Line III shows that the epigenetically mutated DNA methylation in DMR of the ICR leads to the silencing of lncRNA expression. Line IV: small deletion within the ICR. **(C)** Sno-lncRNAs transcribed from paternal allele can recruit Fox proteins and other related proteins, regulating Fox protein distribution and related alternative splicing functions. However, in PWS patients, loss of the *UBE3A-ATS* and other noncoding gene expression lead to the accumulation of Fox proteins in the nucleus and global abnormal splicing patterns. **(D)** Imprinting disorders occur in AS. Line I: deletions of the maternal imprinted regions containing the *UBE3A* and surrounding genes; Line II: both alleles are inherited from paternal chromosome; Line III: *UBE3A* mutations lead to transcript loss of function; Line IV: epimutations in the maternal allele lead to lncRNA expression from the maternal allele, preventing normal *UBE3A* expression.

Recent RNA-Seq data revealed strong allele-biased expression in the adult mouse brain, especially in imprinted regions (Perez et al., 2015, 2016), where many of these genes are expressed in cell type-specific manners. Importantly, mutations or disruptions in imprinted genes are linked with extensive neurobehavioral phenotypes, demonstrating that brain-specific imprinted genes may play important roles in neurodevelopmental disorders (Tucci et al., 2019). PWS and AS are two neurodevelopmental disorders caused by oppositely inherited deficiencies occurred in the same imprinted cluster (Peters, 2014; Kalsner and Chamberlain, 2015; Buiting et al., 2016). These two syndromes perform common phenotype characters, including hypotonia at the newborn stage, abnormal sleep patterns, and the deficiency in intellectual development (Buiting, 2010; Kalsner and Chamberlain, 2015). Children affected by PWS exhibit poor suck phenotypes with reduced muscle tone and mental abilities (Buiting et al., 1995; Buiting, 2010; Fontana et al., 2017), while AS is characterized by deficient motor function, intellectual development, and speech abilities (Buiting, 2010; Eggermann et al., 2015). These two disorders are caused by imprinting disorder in the imprinted PWS/AS locus (*UBE3A/UBE3A-ATS* imprinted cluster) on human chromosome 15q11-13 (Figure 4A). Similar to the mouse homologous locus mentioned previously in Section 3, the E3 ubiquitin ligase-encoding *UBE3A* gene is specifically imprinted in the brain (Vu and Hoffman, 1997). On the maternal allele, the methylated DMR encompasses the promoter of the *SNRPN* gene, silencing the *SNURF/SNRPN* gene and a series of downstream non-coding RNA genes (Rougeulle et al., 1997). In contrast, actively expressed *UBE3A-ATS* and the non-coding *SNORD* gene clusters are expressed from the paternal allele.

On the paternal allele of imprinted human PWS/AS locus, the unmethylated PWS-ICR is the region upstream to a protein-coding gene *SNRPN* and a lncRNA *SNHG14* (small nucleolar RNA host gene 14) (Sutcliffe et al., 1994; Buiting et al., 1995; Rougeulle et al., 1997; Runte et al., 2001; Vitali et al., 2010; Chamberlain, 2013; Stanurova et al., 2018; Figure 4A). The neuron-specific non-coding transcript *SNHG14* is processed to give rise to a series of non-coding RNA products, such as repeated C/D box small nucleolar RNAs (snoRNAs) and lncRNAs including *116HG*, *115HG*, and the antisense transcript to *UBE3A* (Mendiola and LaSalle, 2021; Figure 4A). The most studied RNA product from the host transcript *SNHG14* is *SNORD116* snoRNA, embedded within intronic regions of *SNORD116* gene locus (Cavaillé et al., 2000; de los Santos et al., 2000; Stanurova et al., 2018; Mendiola and LaSalle, 2021). *SNORD116* snoRNA present in ribonucleoprotein complexes (snoRNPs) and may participate in splicing, ribosomal RNA maturation, RNA modifications, and regulation of prohormone processing-related gene expression (Bazeley et al., 2008; Burnett et al., 2017). Meanwhile, *SNORD116* locus encoded *116HG* lncRNA was discovered recently (Vitali et al., 2010). *116HG* is stably retained in the nucleus ‘RNA cloud’ at its transcription site (Powell et al., 2013a). *116HG* potentially regulates transcript levels of circadian-related genes in the cortex and energy-related metabolism through in a time-of-day-dependent manner (Coulson et al., 2018b). Similarly, *SNORD115* locus encodes lncRNA *115HG* and *SNORD115* snoRNAs. While on the maternal allele, the methylated PWS-ICR occurs the upstream of the *SNRPN* gene. It silences the expression of the paternally expressed transcripts while allows the expression of *UBE3A* (Vu and Hoffman, 1997).

Prader–Willi syndrome is the first human disease identified to be caused by the abnormal expression of non-coding RNAs (Sahoo et al., 2008; de Smith et al., 2009; Duker et al., 2010). All cases of PWS in humans involve a deletion in the *SNORD116* non-coding gene locus, which regulates the maturation of the central nervous system. The overlap between the phenotype caused by *SNORD116* microdeletion and *MAGEL2* mutation suggests that transcripts from *SNORD116* locus may modify *MAGEL2* expression via long-range chromatin interactions (Meziane et al., 2015; Fountain and Schaaf, 2016; Langouët et al., 2018). The loss of the paternal expressed *SNORD116* in PWS can be caused by several factors, including large paternal deletions in the imprinted PWS/AS locus (60%), maternal UPD (36%), small microdeletion in *SNORD116* locus (<1%), and epigenetic alternations in DNA methylation of the PWS-ICR region (4%) (Sahoo et al., 2008; Duker et al., 2010; Bieth et al., 2015; Rozhdestvensky et al., 2016; Mendiola and LaSalle, 2021; Figure 4B). Rare microdeletions that encompass *SNORD116* and its adjacent genes, *SNRPN* or *SNORD115*, have been found in PWS patients (Sahoo et al., 2008; de Smith et al., 2009; Duker et al., 2010). Moreover, a small deletion that only covers *SNORD116* and its adjacent genes (*SNORD109A*, and *IPW*) was identified in a patient with typical PWS syndrome (Bieth et al., 2015; Figure 4A). Since there is no obvious involvement of *SNORD109A* and *IPW* genes in PWS, the observations in this PWS case further support that the *SNORD116* gene region play key roles in the PWS, independent with *SNORD115* or *SNRPN* deletion. Consistently, *SNORD116* is completely silenced in neuron cells derived from PWS patients (Cavaillé et al., 2000; Hsiao et al., 2019). Besides, *Snord116* deleted mouse model recapitulates major phenotypes of human PWS patients, including altered metabolism, growth deficiency, memory impairment, hyperphagia and increased anxiety (Skryabin et al., 2007; Ding et al., 2008; Zieba et al., 2015; Qi et al., 2016; Polex-Wolf et al., 2018; Adhikari et al., 2019).

Furthermore, an alternative RNA species (sno-lnRNAs) processed from *SNORD116* host non-coding transcript has been described in human (Yin et al., 2012; Powell et al., 2013a; Figure 4A III). The role of *SNORD116* sno-lncRNAs in RNA processing and decay of their target mRNAs is not well-understood but may facilitates our understanding of the connection between imprinting disorder and pathological mechanism of PWS (Figure 4C). *SNORD116* exon transcript is retained between two snoRNAs, forming sno-lncRNAs with two small nucleolar ribonucleoprotein ends (Yin et al., 2012). These sno-lncRNAs accumulate near the synthesis site together with a type of lncRNAs that are 5′ capped by snoRNAs and 3′ polyadenylated (SPAs) (Wu et al., 2016). These lncRNAs may interact with RNA binding proteins including TDP43 (TAR DNA-binding protein 43), RBFOX2 (RNA Binding Fox-1 Homolog 2), and hnRNP M (Heterogeneous nuclear ribonucleoprotein M). Especially, splicing regulator RBFOX2 are required for the neuron-specific splicing of *Snord116* transcript to produce *116HG* lncRNA and *Snord116* snoRNA (Yeo et al., 2009; Coulson et al., 2018a). Since immunoprecipitation coupled with high-throughput sequencing (CLIP-seq) and RT-PCR assays confirmed that RBFOX2 directly binds to *Snord116* snoRNA, it is hypothesized that *Snord116* snoRNA may reduce the availability of these splicing-related proteins and regulate alternative splicing in the nucleus (Yin et al., 2012; Wu et al., 2016). Therefore, the disruption of *SNORD116* in PWS may lead to more uniform distribution of RBFOX2 protein and global changes in normal alternative splicing patterns, contributing to PWS phenotypes.

In contrast to the paternal-allelic imprinting disorder in PWS, AS, is mainly caused by the lack of maternal *UBE3A* gene expression (Figure 4D; Buiting et al., 2016). The brain-specific and maternally biased expression of *UBE3A* has been shown to function in regulating dendritic growth and influencing behavior and neurotransmitters (Avagliano Trezza et al., 2019). In AS patients, the expression of *UBE3A* or functional UBE3A protein is lost. These alternations can be caused by various imprinting disorder mechanisms including deletions of the maternally imprinted regions containing the *UBE3A* and surrounding genes. Besides pathological variants in the *UBE3A* gene, loss of *SNURF* DMR methylation has also been observed in AS cases (2–3%), in which the expression of *UBE3A* is silenced by *UBE3A-ATS* as discussed previously (Dagli et al., 1993).

#### *KCNQ1OT1* and *H19*/*IGF* in Beckwith–Wiedemann Syndrome and Silver–Russell Syndrome

**FIGURE 5:**
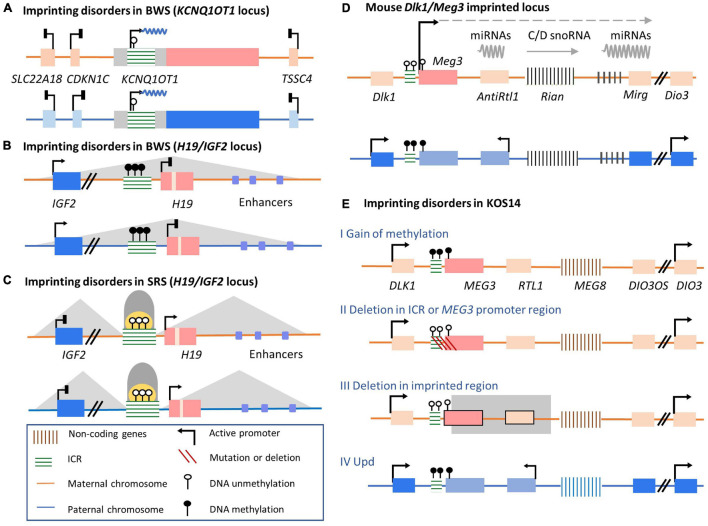
Well-studied imprinting clusters and their conditions in disorder conditions involved in human imprinting disturbance-related diseases. **(A,**B) BWS and two related imprinted clusters, *H19/IGF2* and *KCNQ1OT1*. **(A)** The first situation is the hypomethylation of the maternal allele in *Kcnq1ot1* ICR leads to lncRNA *KCNQ1OT1* overexpression. The expression of neighboring imprinted genes, such as *SLC22A18*, *CDKN1C*, and *TSSC4*, is bi-allelically silenced. **(B)** The second major imprinting disorder responsible for BWS is hypermethylation of the maternal ICR, resulting in loss of *H19* expression and *IGF2* overexpression. **(C)** SRS and alterations in imprinting of *H19/IGF2* locus. Hypomethylation of the paternal *H19/IGF2* ICR resulting in *H19* overexpression and inhibited *Igf2* expression. **(D)** The regulation of mouse *Meg3* imprinted cluster. On the paternal allele, the gDMR of *Meg3* cluster ICR is methylated, repressing *Meg3* lncRNA expression. On the maternal allele, lncRNAs are transcribed from the promoter within the unmethylated ICR. **(E)** Four cases of *MEG3-*related imprinting disorders in KOS14 patients are shown. Line I, epimutations in normally activated maternal ICR of *MEG3* cluster result in loss of lncRNA transcription, releasing normally silenced adjacent imprinted genes; Line II: maternal deletion in ICR of *MEG3* regions; Line III: maternal deletion in the *MEG3* gene body; Line IV: both alleles are inherited by silenced paternal allele.

Beckwith–Wiedemann syndrome and SRS are clinically opposite growth-affecting disorders (Õunap, 2016). The underlying pathological mechanisms involve genetic and epigenetic perturbations of two imprinting clusters on human chromosome 11p15, the *KCNQ1/KCNQ1OT1* and *H19/IGF* loci (Carli et al., 2020; Chang and Bartolomei, 2020; Figures 5A–C). BWS is one of the most common congenital overgrowth conditions (Mussa et al., 2013), with common phenotypes including postnatal overgrowth, placenta mesenchymal dysplasia, and congenital and childhood cancer predisposition. In contrast, SRS patients exhibit postnatal growth failure with body hemihypoplasia, lower birth weight, fetal undergrowth and poor feeding predisposition (Wakeling et al., 2017).

Approximately 50% of BWS patients lose DNA methylation accompanied by loss of H3K9me2 on maternal KvDMR1 (Figure 5A; Robertson, 2005). This epigenetic disturbance results in biallelic expression of the *KCNQ1OT1* lncRNA. As a consequence, expression of this lncRNA silences adjacent imprinted genes on both alleles (Soejima and Higashimoto, 2013). Among these silenced genes, *CDKN1C* is linked to the development of BWS phenotypes (Yan et al., 1997; Zhang et al., 1997; Tunster et al., 2011). These epigenetic mutations in maternal KvDMR1 and biallelic expressed *KCNQ1OT1* lncRNAs lead to loss of CDKN1C expression and fetal overgrowth, thus contributing to BWS syndrome (Eggermann et al., 2016; Wakeling et al., 2017). Therefore, after the establishment of DMRs on imprinted alleles, monoallelic expression of *KCNQ1OT1* lncRNA is a crucial regulator of adjacent protein-coding genes, which have essential roles in maintaining normal growth processes during early development. Another major abnormal imprinted cluster identified in BWS patients is *H19/IGF2* (Figure 5B). Under normal conditions, *H19/IGF2* ICR is methylated on the paternal chromosome, controlling the expression of *H19*. In BWS patients, mutations or hypermethylation of the *H19/IGF2* ICR can lead to *H19* silencing and subsequent overexpression of *IGF2*, a circulating hormone and tissue growth factor. The upregulated expression of *IGF2* is linked to BWS overgrowth-related phenotypes (Pollak et al., 2004; Brioude et al., 2018a, b; Duffy et al., 2019). As for SRS, loss of *H19/IGF2* ICR methylation on the paternal chromosome 11p15 accounts for 40–60% of patients (Wakeling et al., 2017). ICR hypomethylation is bound by the insulator CTCF. The interaction of the *IGF2* promoter with its enhancer on both alleles is disrupted, resulting in decreased *IGF2* expression and subsequent growth and development delays (Figure 5C; Abi Habib et al., 2017).

Although some BWS and SRS patients can be identified based on clinical features alone, diagnosing imprinting disorders can be complicated by complex molecular alternations (Ibrahim et al., 2014; Wakeling et al., 2017). In addition to the two imprinted loci primarily relevant to BWS and SR phenotypes, MLID has also been observed in an increasingly growing fraction of patients with methylation abnormalities at other imprinted loci (Rossignol et al., 2006; Azzi et al., 2009; Eggermann et al., 2011; Fontana et al., 2018). In addition, symptoms vary widely in patients with imprinting disorders (Wakeling et al., 2017; Brioude et al., 2018b; Mantovani et al., 2018). Therefore, additional insights into the relationship between the epigenetic mechanisms of imprinting disorders and neurological diseases can help clarify more accurate diagnostic guidelines and appropriate clinical therapies.

#### *DLK1/DIO3* in Kagami–Ogata Syndrome and Temple Syndrome

Genetic and epigenetic alterations in delta-like homolog 1 gene/type III iodothyronine deiodinase gene (*DLK1/DIO3*) imprinted cluster on human chromosome 14q32 are associated with two human imprinting disorder-related diseases, KOS14 and TS14 (Temple et al., 1991; Wang et al., 1991; Ogata and Kagami, 2016). Common KOS14 phenotypes include neonatal respiratory difficulties, a distinctive facial appearance, variable developmental delay, and/or intellectual disability (Ogata and Kagami, 2016; Prasasya et al., 2020). Clinical syndromes observed in TS14 include severe intrauterine growth restriction, postnatal growth restriction, neonatal hypotonia, and feeding difficulties in infancy (Ioannides et al., 2014; Gillessen-Kaesbach et al., 2018; Prasasya et al., 2020).

The distribution of imprinted genes and regulatory mechanisms of *DLK1/DIO3* locus are highly conserved between humans and mice. The regulation of this imprinted locus has been revealed in mouse models established with genetic alterations in the *Dlk1/Dio3* locus on chromosome 12 (Figure 5D; Paulsen et al., 2001; da Rocha et al., 2008). Three paternally expressed imprinted protein-coding genes are *Dlk1*, *Rtl1*, and *Dio3*. lncRNA *Meg3* (also called *Gtl2*), the *Rtl1*-antisense *Rtl1as*, the C/D-box snoRNA cluster *Rian*, and the microRNA cluster *Mirg* are transcribed from the maternal allele (da Rocha et al., 2008; Kota et al., 2014). The regulation of imprinted gene expression in this locus relies on an intergenic DMR (IG-DMR). On the maternal allele, AFF3 protein binds to an upstream enhancer of *Meg3*, activating lncRNA expression. In contrast, on the paternal allele, AFF3 binds instead to the methylated IG-DMR, leading to silencing of *Meg3* and other non-coding genes (Luo et al., 2016; Wang et al., 2017). It has also been suggested recently that maternally expressed lncRNA *Meg3* is involved in the regulation of the *Dlk1/Dio3* imprinted cluster (Sanli et al., 2018). The maternal expression of the *Meg3* lncRNA may play a role in preventing maternal *Dlk1* activation through interaction with the lysine methyltransferase (KMT) Ezh2 and PRC2 in the maternal *Dlk1* gene region (Kaneko et al., 2014; Sanli et al., 2018). Remarkably, *Meg3* lncRNA’s regulation of imprinted protein-coding gene *Dlk1* is restricted to a developmental window as follows. In embryonic stem cells, the *Dlk1* gene is expressed biallelically at a low level. Upon neuronal differentiation, *Dlk1* expression is upregulated on the paternal allele. Conversely, the activation of the *Dlk1* gene on the maternal allele is prevented by the overlap of *Meg3* lncRNA *in cis* and the recruitment of Ezh2 to the *Dlk1* gene region (Sanli et al., 2018). Although the *Meg3* lncRNA is necessary for the silencing of *Dlk1* expression, the mechanisms underlying the connection between the *Meg3* lncRNA and repressed *Dlk1* expression on the maternal allele are unknown.

The *DLK1/DIO3* locus is predominantly imprinted in the human brain (Davis et al., 2005; Ferrón et al., 2011). Protein-coding genes *DLK1*, *RTL1*, and *DIO3* are expressed on the paternal allele; lncRNAs (*MEG3*, *MEG8*, *RTL1as*, *DIO3OS*), snoRNAs, and miRNAs are transcribed on the maternal allele. Importantly, the *DLK1* gene plays essential functions in regulating development and metabolism. In KOS14 patients, gain of DNA methylation on the maternal ICR leads to *MEG3* silencing (Figure 5E I; Sato et al., 2011). However, maternal micro-deletions of the *MEG3* promoter that don’t affect ICR methylation are also observed in some cases (Figure 5E II; Kota et al., 2014). In another case, a maternal micro-deletion has been detected in the *MEG3* gene body instead of the IG-DMR or *MEG3* promoter (Figure 5E III; van der Werf et al., 2016).

In summary, in these conditions, imprinted lncRNAs play essential roles as upstream regulators of protein-coding genes in the same imprinted cluster. However, the detailed mechanisms are diverse and complicated in different imprinting disorders and remain to be further investigated.

### Imprinted Long Non-coding RNAs and Human Cancers

Long non-coding RNAs play important roles in pathways implicated in many cancer types, including prostate (Hua et al., 2018, p. 19), breast (Zhang et al., 2017; Cho et al., 2018), and hepatocellular carcinoma (He et al., 2017; Lecerf et al., 2019; Ye et al., 2020). Long non-coding RNAs can serve as cancer enhancers or repressors in temporal- and spatial-specific manners (Calin et al., 2007; Kanduri, 2016; Quinn and Chang, 2016; Peng et al., 2017). Abnormal functions of lncRNAs have been observed in various tumors and cancer cell lines (Kitagawa et al., 2012; Bhan et al., 2017). Notably, abnormally regulated imprinted gene expression, altered ICR methylation conditions, and altered expression of cancer-related imprinted lncRNAs were observed in cancers such as breast cancer (Kim et al., 2015; Goovaerts et al., 2018). In addition, in imprinting disorders, abnormal silencing of imprinted lncRNAs contributes to congenital and childhood tumors. For instance, susceptibility to Wilm’s tumor and adrenocortical carcinoma is increased in *H19*-silenced patients (Dao et al., 1999; DeBaun et al., 2000; Weksberg et al., 2010; Brioude et al., 2018a, b).

*H19* is one of the most commonly implicated tumorigenesis-promoting lncRNAs (Zheng et al., 2020). The expression of *H19* occurs during embryonic development and decreases after birth in most tissues. However, *H19* is abnormally upregulated in various cancers, including breast, liver, lung, esophageal, pancreatic, ovarian, and bladder (Vennin et al., 2015; Zhang et al., 2016). *H19*’s tumor-promoting effects include the inhibition of cell death, promotion of proliferation, downregulation of growth suppressors, and promotion of invasion and metastasis (reviewed in Matouk et al., 2015; Lecerf et al., 2019). Moreover, high *H19* expression may be a molecular marker to predict cancers and prognoses after clinical treatment, including the rate of post-therapeutic relapse in hematological cancer patients (Liu et al., 2016). Increased risk of developing congenital and childhood tumors seen in BWS is also associated with aberrant *H19*. *H19* is also associated with growth suppression (Yoshimizu et al., 2008; Lecerf et al., 2019; Zhou et al., 2019). *H19*’s contribution to tumorigenesis varies by tissue and developmental windows and requires clarification in future investigations.

Another well-studied cancer-related imprinted lncRNA is *MEG3*, which acts as a cancer repressor. *MEG3* is downregulated in breast, neuroblastoma, meningioma, glioma, pituitary adenoma, and hematological malignancies (Benetatos et al., 2011; Cheunsuchon et al., 2011; Zhou et al., 2012; Lyu et al., 2017; Zhu et al., 2019). In pituitary neuroendocrine tumors, hypermethylation of the maternal *DLK1/MEG3* locus results in *MEG3* downregulation and impaired differentiation (Cheunsuchon et al., 2011; Chen et al., 2020). Hypermethylation of the *MEG3* promoter region has also been observed in AML patients (Lyu et al., 2017; Yao et al., 2017; Sellers et al., 2019), while recent studies have begun to reveal the underlying mechanisms in endometrial and breast cancers (Sun et al., 2017; Zhang et al., 2017; Zhu et al., 2019). One such mechanism involves *MEG3*’s inhibition of the phosphoinositide 3-kinase/protein kinase B (*PI3K/Akt*) signaling pathway, a well-known growth-related pathway. Therefore, unraveling the roles of imprinted lncRNAs in cancer may reveal novel biomarkers and therapeutic targets for cancer treatment.

## Modulation of the Long Non-Coding RNA *Ube3A-ATS* to Rescue Abnormal Imprinting in Prader–Willi Syndrome/Angelman Syndrome Imprinted Cluster

Although our understanding of the mechanisms of imprinting disorders has grown, efficient molecular diagnosis and effective treatments are limited to nonexistent (Elbracht et al., 2020). Modulation of imprinted lncRNAs has been proposed as a potential therapeutic strategy to target imprinted genes and rescue imprinting disorders (Peters, 2014; Statello et al., 2021). As the epigenetic regulatory mechanisms of the *Ube3a/Ube3a-ATS* imprinted cluster are understood best, attempts have been made to rescue *Ube3a* expression through modulating the collision between the transcriptional machinery of *Ube3a* and *Ube3a-ATS* in an allele-specific manner. Herein, three state-of-the art therapeutic strategies by targeting *Ube3a-ATS* lncRNA, editing *Ube3a-ATS* gene region, or modulating chromatin transcriptional state by small molecules are discussed along with recent preclinical studies of *UBE3A/UBE3A-ATS* imprinted cluster-related diseases.

### Antisense Oligonucleotides for Imprinted Long Non-coding RNAs

Antisense oligonucleotides are single-stranded DNA oligos designed using sequence homology with their RNA targets that hybridize with the targeted RNA region based on complementary base pairs, and induce subsequent RNA degradation at the ASO-RNA heteroduplex part (Mishra et al., 2019; Li M. et al., 2020). ASOs can be used to alter splicing or gene expression. ASOs have been designed as potential therapies for various diseases, including AS, spinal muscular atrophy (SMA), Duchenne muscular dystrophy, Huntington disease, and hyperlipidemia (Beaudet and Meng, 2016; Dhuri et al., 2020). Several ASO-based therapies, such as Nusinersen (Spinraza) for SMA treatment, have received approval by the United States Food and Drug Administration (FDA) and other regional regulatory agencies (Karaki et al., 2019). Nusinersen is quite effective in rescuing protein deficiency by altering pre-mRNA splicing (Hoy, 2017; Groen et al., 2018; Claborn et al., 2019). The capacities of ASOs to access targeted RNAs through homology base pairing and in inducing RNase H-mediated cleavage at the pairing regions by exonucleases make them suitable to decrease lncRNA levels post-transcriptionally (Chan et al., 2006).

**FIGURE 6:**
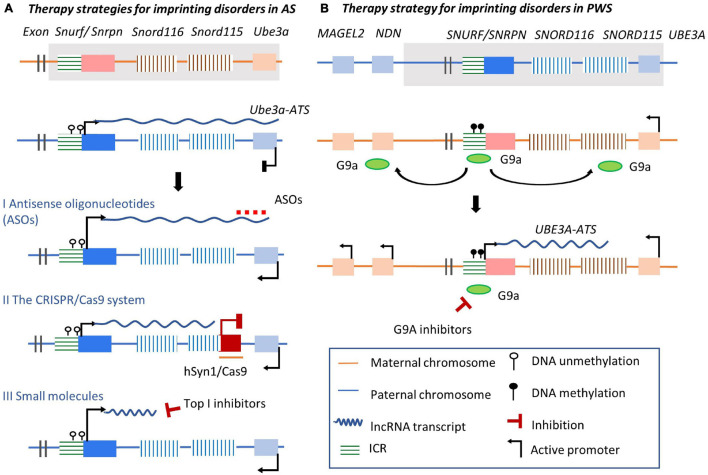
Three state of the art strategies for imprinted disorders via targeting imprinted lncRNAs. **(A)** Therapeutic strategies for AS. Molecular alterations such as deletions in *Ube3a/Ube3a-ATS* imprinted cluster can cause the loss of effective *Ube3a* expression. Line I: ASOs are designed to target the overlapping regions of *Ube3a-ATS* transcripts and *Ube3a*, releasing paternal *Ube3a* expression; Line II: the human synapsin 1 (hSYN1) gene promoter is used drive neuron-specific expression and Cas9 packaged with adeno-associated virus delivering system is inserted into the gene region of the *Snord115*, leading to disrupted transcription of *Ube3a-ATS* before extending to the *Ube3a* gene encoding region. Line III: Top I inhibitors disrupt the elongation of the *Ube3a-ATS* at the *Snord116* region. *Ube3a* paternal expression is released from transcriptional collision. **(B)** Therapeutic strategy for PWS. G9A inhibitors prevent G9A recruitment to flanking regions near the ICR, releasing *Ube3a-ATS* lncRNA expression from its promoter in the ICR.

As mentioned before, on the paternal allele of *Ube3a/Ube3a-ATS* imprinted cluster, *Ube3a-ATS* represses *Ube3a* expression by prematurely terminating the elongation of *Ube3a* transcripts. Therefore, a potential strategy is to rescue the defective *Ube3a* transcription by targeting *Ube3a-ATS* transcripts using ASOs (Figure 6A I). To avoid influencing the transcription of sno-lncRNAs essential for neuronal development and PWS, ASOs were designed to be complementary to *Ube3a-ATS* transcripts downstream of the *Snord115* cluster. These ASOs were provided to cultured AS mouse neurons with deficient *Ube3a* expression (Meng et al., 2015). The treatment achieved sustained ectopic paternal expression of *Ube3a*, partially rescued *UBE3A* brain protein levels, and alleviated some cognitive deficits. Remarkably, other splicing products derived from *Ube3a-ATS* like *Snrpn* and *Snord116* were unaffected. Consistently, ASOs were designed to rescue the expression of *UBE3A* in AS iPSC-derived neuron cells with a large deletion of maternal 15q11-q13. ASOs targeting *UBE3A-ATS* transcripts at *SNORD115* and *SNORD109B*, or targeting the snoRNA located between *SNORD 115* locus and *UBE3A* gene region, cleave *UBE3A-ATS* and release the transcription of *UBE3A* on the paternal chromosome (Germain et al., 2021). *UBE3A-ATS* transcription is terminated by displacing RNA Polymerase II several kilobases downstream of the ASO targeting site. Therefore, targeting the lncRNA *UBE3A-ATS* by ASOs could be a potential strategy for rescuing *UBE3A* expression and related imprinting disorders. Besides, ASOs have several unique features in treating imprinted disorders, including high *in vivo* efficacy, broad tissue distribution, low adverse events, and long duration of action (Smith et al., 2006; Kordasiewicz et al., 2012). Considering that several mRNA-targeting ASOs have been approved (Dhuri et al., 2020), targeting lncRNAs using ASOs to treat imprinting diseases could achieve wide application. However, robust delivery systems devoid of associated toxicity should be carefully developed and evaluated.

### Modulation of Imprinted Long Non-coding RNA Expression Using the CRISPR/Cas9 System

The CRISPR/Cas9 system permits *in vitro* and *in vivo* gene editing tool and is another novel strategy to modulate imprinted lncRNA expression (Deltcheva et al., 2011; Jinek et al., 2012; Konermann et al., 2015; Suzuki et al., 2016; Knott and Doudna, 2018). A series of CRISPR/Cas-engineered systems can be designed to manipulate lncRNAs, including deletion of the lncRNA encoding gene region (pre-transcription level), inhibition or activation of the expression of the lncRNA (transcription level), or direct degradation of the lncRNA transcripts (post-transcriptional level) (Perez-Pinera et al., 2013; Qi et al., 2013; Ran et al., 2013; Abudayyeh et al., 2017). For example, CRISPRi and CRISPRa can modulate lncRNA expression by recruiting transcriptional repressors or activators without inducing genetic mutations (Bester et al., 2018; Kampmann, 2018). At the same time, CRISPR/Cas9 is being studied as a strategy of *in vivo* genome editing therapy in neurological diseases like schizophrenia and Alzheimer’s disease (Zhuo et al., 2017; Kuruvilla et al., 2018; Park et al., 2019; Sun et al., 2019). It is hoped that effective manipulation of the non-coding regions achieved in human cell lines and animal models could result in novel strategies to eliminate obstacles in developing therapies for lncRNA-related imprinting diseases (Cho et al., 2013; Cong et al., 2013; Jiang et al., 2013; Doudna and Charpentier, 2014, p. 9). Furthermore, when taking into account brain-specific expression of imprinted clusters, CRISPR/Cas9 could be designed to correct abnormal imprinting patterns (Han et al., 2014). Indeed, recently Cas9 gene therapy has shown promise in trapping *Ube3a-ATS* to activate paternal Ube3a expression (Figure 6A II; Wolter et al., 2020). In addition, a CRISPR/Cas9 system targeting the *Snord115* locus in cultured mouse cortical neurons and human neural progenitor-derived neurons was able to successfully increase total *Ube3a* protein expression while decreasing *Snord115* expression. Using a neuron-specific saCas9 and guide RNAs packaged in an adeno-associated virus delivering system and administered to an AS mouse brain during the embryonic and early postnatal stages led to silencing of paternal *Snord115* expression with long-lasting effects.

In summary, the CRISPR-Cas9 system offers promising therapeutic strategies with the potential to permanently alter imprinted gene expression with high specificity and low toxicity. Nevertheless, since lncRNAs lack open reading frames and functional protein products, the use of CRISPR-Cas9 system to achieve efficient lncRNA manipulation needs to be further improved (Statello et al., 2021). In addition, an optimal sgRNA design and an effective delivery mechanism to penetrate the blood-brain barrier need further investigation (Zhuo et al., 2017; Hana et al., 2021).

### Small Molecules Targeting Histone Modifiers

Small molecules have been screened to target histone modification proteins involved in imprinted lncRNA regulation. As mentioned before, PWS and AS are two imprinting disorders related to the same imprinted cluster. In AS patients, *UBE3A* expression is decreased. Through high-content screening in mouse-derived primary cortical neurons, about 10 topoisomerase I (Top I) inhibitors have been identified with the capacity to downregulate *Ube3a-ATS* expression and induce reactivation of *UBE3A* expression from the paternal allele (Huang et al., 2011; Powell et al., 2013b). The Top I inhibitor topotecan blocks the elongation of the *Ube3a-ATS* transcription complex in cultured mouse neurons (Powell et al., 2013b). It inhibits sno-lncRNA transcription throughout the *Ube3a* encoding gene region by stabilizing the formation of R loops between RNA and DNA within paternal *Snord116*, leading to chromatin decondensation (Liu and Wang, 1987; Belotserkovskii et al., 2010; El Hage et al., 2010; Belotserkovskii and Hanawalt, 2011; French et al., 2011; Skourti-Stathaki et al., 2011; Aguilera and García-Muse, 2012; Ginno et al., 2012; Figure 6A III). The *Ube3a-ATS* transcription complex stalled before transcription of Sno-lncRNAs completed. Subsequent *Ube3a* expression was reactivated on the paternal allele. Additional candidates of other Top I inhibitors have also been assessed to identify inhibitors with better pharmacological profiles of *Ube3a* activation (Lee et al., 2018). Prospective therapeutic safety and central nervous system (CNS) bioavailability studies have also been performed recently in AS mouse neurons (Lee et al., 2018).

A therapeutic strategy for PWS based on the induction of *SNORD116* expression has been proposed. *SNORD116* is normally silenced on the maternal allele, but its expression can be induced by modulating ‘closed’ chromatin condition into an ‘open’ state (Kim et al., 2017). The methylation of histone H3K9 performs allele-specific pattern in the ICR located upstream of *SNRPN* gene (PWS-ICR). On the maternal chromosome, histone methyltransferase euchromatic histone lysine N-methyltransferase-2 (G9a) locates at the methylated PWS-ICR and recruits repressive histone modifications (H3K9me2) along the PWS-ICR in a bidirectional manner. This leads to condensed chromatin structure and silencing of PWS-associated genes (Figure 6B). The inactivation of histone H3K9 methyltransferase G9a in mouse embryonic stem (ES) cells leads to reduced DNA methylation in PWS-ICR, and the expression of *Snrpn* was activated on both chromosome *in vitro* (Xin et al., 2003). However, in *in vivo* mouse model, two inhibitors of G9a selected lead to the activation of maternal copy of *Snord116* and improve survival of the PWS mouse without effect on the methylation state of the PWS-ICR or *Ube3a* expression on the maternal allele (Kim et al., 2017). Thus, further studies are needed to clarify the association between DNA methylation of PWS-ICR and allele-specific distribution of G9a. Meanwhile, the reactivation of *SNRPN* and *SNORD116* was recently achieved by preventing the recruitment of H3K9me3 repressive histone modification-related protein factor to *SNORD116* locus in PWS-derived iPSCs (Langouët et al., 2020). In summary, small molecules related epigenetic therapy for PWS through modulating the condition of specific chromatin regions could be a potential strategy to be translated in clinical relevance (Crunkhorn, 2017; Chung et al., 2020).

## Concluding Remarks

Several imprinting gene clusters have been discovered and studied since the middle of the last century. These studies have shown that lncRNAs play crucial roles in regulating imprinted gene clusters and individual imprinted genes related to human health and diseases. However, from a genomic perspective, the characteristics of gene regulation among imprinting loci remain to be fully elucidated. This is despite the advancement in knowledge of the epigenetic regulatory mechanisms of a subset of genes in imprinted regions. In the three imprinted clusters (*Airn/Igfr2*, *Kcnq1/Kcnq1ot1*, and *Ube3a/Ube3a-ATS*), imprinted lncRNAs which play essential regulatory roles in silencing other imprinted genes are all expressed on the paternal allele. It has been reported that maternal expressed imprinted genes are prominent with protein-coding genes, while paternal expressed genes exhibit consistent distribution between non-coding and protein-coding sequences (Hutter et al., 2010). However, the difference between the establishment of maternal and paternal imprinted genes in lncRNA mechanisms remains unclear. Thus, comprehensive investigations are needed to understand further the mechanisms of imprinted lncRNAs in the epigenetic regulation of imprinted clusters. With technological advancements, studies on lncRNA-associated human imprinting disorders will lead to needed therapies.

Pharmacological treatments for congenital imprinting disorders are limited to symptomatic therapies, which are inefficient in promoting the patients’ quality of life (Chung et al., 2020). Fortunately, the biological role of lncRNAs in the etiology of congenital imprinting disorders has been revealed thanks to the advancement in high-throughput genome-wide sequencing technologies. Therapeutic approaches based on disease-related lncRNAs have been investigated. In a recent study, lncRNA mimics were designed to restore the tissue-specific lncRNA *HULC* in mice, essential for phenylalanine metabolism (Li et al., 2021). In addition, three strategies mentioned above targeting Ube3a-ATS have efficiently rescued imprinting disorders in PWS/AS imprinted cluster in mouse models and human cell lines. Although the three strategies mentioned here targeting Ube3a-ATS have efficiently rescued imprinting disorders of PWS/AS imprinted cluster in mouse models and human cell lines, therapies for other disease-related clusters have not been investigated. Long non-coding RNA-based and lncRNA-targeting therapies have some unique advantages. For instance, in lncRNA-targeting methods like ASOs, synthesized RNA can be designed with organ-targeting peptides to achieve tissue-specific targeting of endogenous lncRNAs. Besides, synthesized RNA products could be modified to promote *in vivo* stability. Further translation of these strategies to real clinical tools will require further investigation to overcome related challenges. *In vivo* delivery of synthesized RNA molecules, cellular permeability, immunogenicity, and potential of organ toxicity also deserve further investigation (Perry and Ulitsky, 2021). Another challenge to extend the lessons learned in PWS and AS into other imprinting disorders is the epigenetic and molecular complexities in different imprinting disorders-related imprinted loci. Considering the complexity of the regulatory network of genomic imprinting, further efforts are needed to reveal underlying pathological mechanisms linked to imprinting disorder phenotypes and support continuous improvement of clinical management and therapeutic strategies.

## Author Contributions

TW and QM wrote and edited the manuscript and drew the pictures. JL, LY, and MW assisted in manuscript collation and review. QM provided critical inputs as the corresponding authors and obtained funds. All authors contributed to the article and approved the submitted version.

## Conflict of Interest

The authors declare that the research was conducted in the absence of any commercial or financial relationships that could be construed as a potential conflict of interest.

## Publisher’s Note

All claims expressed in this article are solely those of the authors and do not necessarily represent those of their affiliated organizations, or those of the publisher, the editors and the reviewers. Any product that may be evaluated in this article, or claim that may be made by its manufacturer, is not guaranteed or endorsed by the publisher.

## Abbreviations

ASE: allele-specific gene expression
MAE: monoallelic expression
PWS: Prader–Willi syndrome
AS: Angelman syndrome
BWS: Beckwith–Wiedemann syndrome
SRS: Silver–Russell syndrome
KOS14: Kagami–Ogata syndrome
TS14: Temple syndrome
ICRs: imprinting control centers or imprinting control regions
gDMRs: germline differentially methylated regions
lncRNAs: long non-coding RNAs
ASOs: antisense oligonucleotides
SNPs: single nucleotide polymorphisms
H4R3me2s: histone H4 arginine-3 symmetrical demethylation
H3K9me3: H3 lysine-9 trimethylation
Igf2: the insulin-like growth factor 2
Kcnq1/Kcnq1ot1: potassium voltage-gated channel subfamily Q member 1/Kcnq1 antisense transcript 1
KvDMR1: KvLQT1 differentially methylated region 1
PRC: polycomb repressive complex
EHMT2: histone methyltransferase euchromatic histone lysine *N*-methyltransferase -2
Airn: the antisense of Igf2r non-protein coding RNA
H2AK119ub1: lysine 119-monoubiquititinated histone H2A
Ube3a-ATS: Ube3a-antisense lncRNA
UPD: uniparental disomy
MLID: multi-locus imprinting disturbance
SNORD116: SnoRNA C/D box cluster 116
RBFOX2: RNA binding protein fox-1 homolog 2
DLK1/DIO3: delta-like homolog 1 gene/type III iodothyronine deiodinase gene
Rtl1as: the Rtl1-antisense
PI3K/Akt: phosphoinositide 3-kinase/protein kinase B
CRISPRi: CRISPR interference
CRISPRa: CRISPR activation
topoisomerase I: (Top I) inhibitors.
